# *n*-6 High Fat Diet Induces Gut Microbiome Dysbiosis and Colonic Inflammation

**DOI:** 10.3390/ijms22136919

**Published:** 2021-06-28

**Authors:** Ornella I. Selmin, Andreas J. Papoutsis, Sabine Hazan, Christopher Smith, Nick Greenfield, Micah G. Donovan, Spencer N. Wren, Thomas C. Doetschman, Justin M. Snider, Ashley J. Snider, Sherry H.-H. Chow, Donato F. Romagnolo

**Affiliations:** 1 Department of Nutritional Sciences, The University of Arizona, Tucson, AZ 85721, USA; selmin@arizona.edu (O.I.S.); spencerwren@arizona.edu (S.N.W.); justinsnider@arizona.edu (J.M.S.); ashleysnider@arizona.edu (A.J.S.); 2 The University of Arizona Cancer Center, Tucson, AZ 85724, USA; schow@arizona.edu; 3 ProgenomaBiome, Ventura, CA 93003, USA; papoutsis@progenabiome.com (A.J.P.); drhazan@progenabiome.com (S.H.); 4 One Codex, San Francisco, CA 94110, USA; christopher@onecodex.com (C.S.); nick@onecodex.com (N.G.); 5 Cancer Biology Graduate Interdisciplinary Program, The University of Arizona, Tucson, AZ 85724, USA; mdono123@arizona.edu; 6 Department of Molecular and Cellular Medicine, The University of Arizona, Tucson, AZ 85724, USA; tdoets@arizona.edu; 7 Department of Medicine, The University of Arizona, Tucson, AZ 85724, USA

**Keywords:** omega-6, fatty acids, gut, dysbiosis, colon, inflammation

## Abstract

**Background**: Concerns are emerging that a high-fat diet rich in *n*-6 PUFA (*n*-6HFD) may alter gut microbiome and increase the risk of intestinal disorders. Research is needed to model the relationships between consumption of an *n*-6HFD starting at weaning and development of gut dysbiosis and colonic inflammation in adulthood. We used a C57BL/6J mouse model to compare the effects of exposure to a typical American Western diet (WD) providing 58.4%, 27.8%, and 13.7% energy (%E) from carbohydrates, fat, and protein, respectively, with those of an isocaloric and isoproteic soybean oil-rich *n*-6HFD providing 50%E and 35.9%E from total fat and carbohydrates, respectively on gut inflammation and microbiome profile. **Methods**: At weaning, male offspring were assigned to either the WD or *n*-6HFD through 10–16 weeks of age. The WD included fat exclusively from palm oil whereas the *n*-6HFD contained fat exclusively from soybean oil. We recorded changes in body weight, cyclooxygenase-2 (COX-2) expression, colon histopathology, and gut microbiome profile. **Results**: Compared to the WD, the *n*-6HFD increased plasma levels of *n*-6 fatty acids; colonic expression of COX-2; and the number of colonic inflammatory and hyperplastic lesions. At 16 weeks of age, the *n*-6HFD caused a marked reduction in the gut presence of *Firmicutes*, *Clostridia*, and *Lachnospiraceae*, and induced growth of *Bacteroidetes* and *Deferribacteraceae*. At the species level, the *n*-6HFD sustains the gut growth of proinflammatory *Mucispirillum schaedleri* and *Lactobacillus murinus*. **Conclusions**: An *n*-6HFD consumed from weaning to adulthood induces a shift in gut bacterial profile associated with colonic inflammation.

## 1. Introduction

During the last two decades, there has been an increase in the incidence of pediatric and adult inflammatory bowel diseases (IBD) [1,2]. In the U.S. alone, ~1.4 million adults suffer from IBD. Genetic factors account for only a modest proportion of the disease, ranging from ~14% for Chron’s disease (CD) to ~7.5% for ulcerative colitis (UC) [3]. Concurrently with recent recommendations to limit the intake of saturated fatty acids (SFA) [4], there has been a marked increase in intake of *n*-6 polyunsaturated fatty acids (PUFA) primarily from vegetable oils rich in 18:2*n*-6 linoleic acid (LA) [5]. However, concerns have emerged that a high-fat diet (HFD) rich in *n*-6 PUFA (*n*-6HFD) exacerbate the risk of intestinal diseases [6,7] and obesity [8]. These concerns also stem from evidence that adherence to other dietary patterns rich in monounsaturated fatty acids (MUFA) and *n*-3 PUFA [9] and lower in *n*-6 PUFA lower the risk of gut inflammation [10]. The latter is a condition that predisposes to colorectal cancer [11,12].

The type and relative amount, and timing and duration of exposure, to dietary fat may influence the risk of gut dysbiosis and intestinal inflammation. For example, compared to a low-fat diet (LFD) providing 10% energy (%E) from fat, a HFD (60%E from fat) rich in *n*-6 PUFA (~30% total fat) augments the number of gram-negative *Enterobacteriaceae* and intestinal inflammation characterized by increased expression of cyclooxygenase-2 (COX-2) and cytokines, and higher intestinal permeability [13]. However, most rodent studies modeling human consumption of *n*-6HFD have focused on short-term feeding (~2–8 weeks) [13,14,15,16,17,18], whereas rodent gut investigations of microbial ecology modeling human consumption of *n*-6HFD by young adult (8–12 weeks) to adult (>12 weeks) [19] are scarce. To address this knowledge gap, the objective of this study was to examine, in a mouse model, the effects of feeding an *n*-6HFD, from weaning to adulthood, on endpoints of colonic inflammation and gut microbiome profile compared to those elicited by a Western diet (WD). The *n*-6HFD was formulated to provide 50%E from fat, of which 28%E was LA from soybean oil. The WD was isocaloric and isoproteic, and designed to model the typical diet of Americans between 12 and 29 years of age, which comprises ~50%E, 35%E, and 15%E from carbohydrates, fat, and protein, respectively, with SFA contributing ~13%E compared to only ~7.5%E from PUFA [20]. The WD included fat exclusively from palm oil whereas the *n*-6HFD contained fat exclusively from soybean oil. We report that, compared to a WD, long-term (16 weeks) consumption of an *n*-6HFD from weaning, induces, in adulthood, changes in the number of inflammatory and preneoplastic lesions in the colon, and in expression of the inflammatory marker COX-2. Moreover, the *n*-6HFD elicits a marked pathobiontic growth in the gut.

## 2. Results

### 2.1. Body Weight and Plasma Fatty Acid Profile

**Figure 1:**
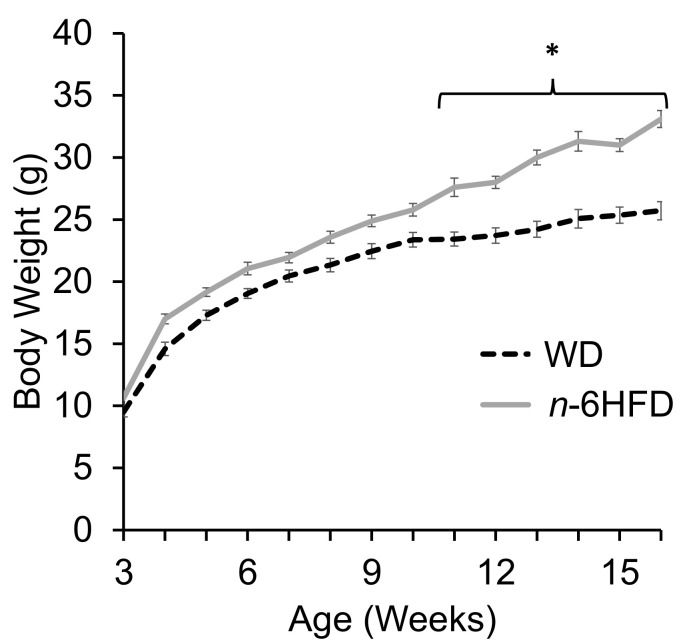
The *n*-6HFD induces higher body weight of male C57BL.J6 mice compared to the WD. Asterisks indicate statistical significance (*p* < 0.05, *n* = 15).

The nutrient and fatty composition of the diets (please see Materials and Methods) show that the WD provided fat exclusively from palm oil whereas the *n*-6HFD contained fat exclusively from soybean oil. By 16 weeks of age, animals on the *n*-6HFD diet gained on average ~25% more weight than those on the WD (Figure 1), with body weight gains between the two groups diverging significantly, starting at 10 weeks and increasing thereafter. These results parallel those of previous feeding studies with HFD in rodent models and similar duration [21].

**Figure 2:**
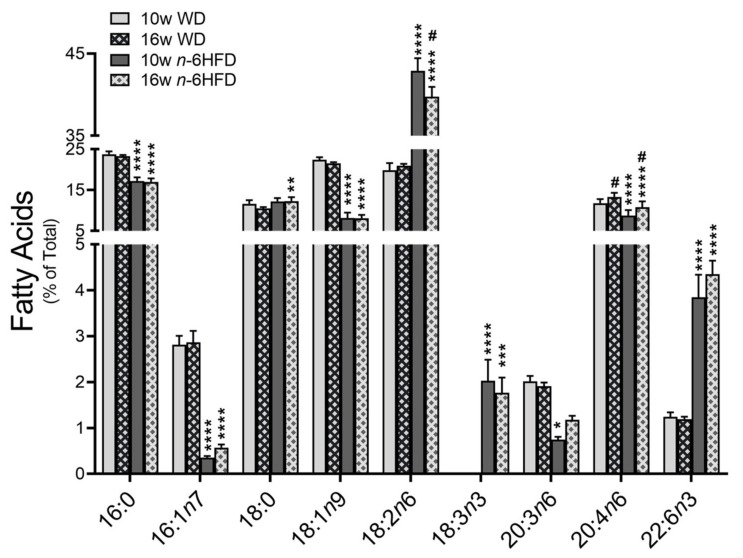
Plasma fatty acid profile in C57BL/J6 male mice fed the WD or *n*-6HFD through 10 and 16 weeks of age. Bars represent average percentage values + SD of total plasma fatty acids (*n* = 5 for each diet at 10 and 16 weeks) for 16:0 palmitic acid; 16:1*n*7 palmitoleic acid; 18:0 stearic acid; 18:1*n*9 oleic acid; 18:2*n*6 linoleic acid; 18:3*n*3 α-linolenic acid; 20:3*n*6, dihomo-γ-linolenic acid; 20:4*n*6 arachidonic acid; and 22:6*n*3 docosahexanoic acid. Data represent mean ± SD; * *p* < 0.05, ** *p* < 0.01, *** *p* < 0.001, and **** *p* < 0.0001 represent statistically significant differences between WD and *n*-6HFD from the same time point; # *p* < 0.01 represents significant differences between time points with the same diet.

Measurements of fatty acids in plasma (Figure 2) show that compared to the WD, feeding of the *n*-6HFD lowers at 10 and 16 weeks the levels of 16:0 palmitic (~24% to 17%), 16:1*n*7 palmitoleic (~2.8% to 0.5%), 18:1*n*9 oleic acid (~22.0% to 8.0%), and 20:4*n*6 arachidonic (~13% to 10%) acid; and at 10 weeks 20:3*n*6 dihomo-γ-LA (~2.0% to 0.8%). Conversely, compared to the WD, the *n*-6HFD increases at 10 and 16 weeks the plasma concentration of 18:2*n*6 LA (~20% to ~40%), 18:3*n*-3 α-linolenic acid (not detectable to ~2.0%), and 22:6*n*-3 docosahexaenoic (~1.2% to ~4.0%); and at 16 weeks the plasma level of 18:0 stearic acid (10% to 12%). Data on other plasma fatty acids for which there were no statistical differences are reported in Figure S1, Supplementary Materials. Overall, these data document important shifts in the plasma fatty acid profile of animals fed the *n*-6HFD compared to mice on the WD.

### 2.2. Histopathological Analysis and COX-2 Expression

**Figure 3:**
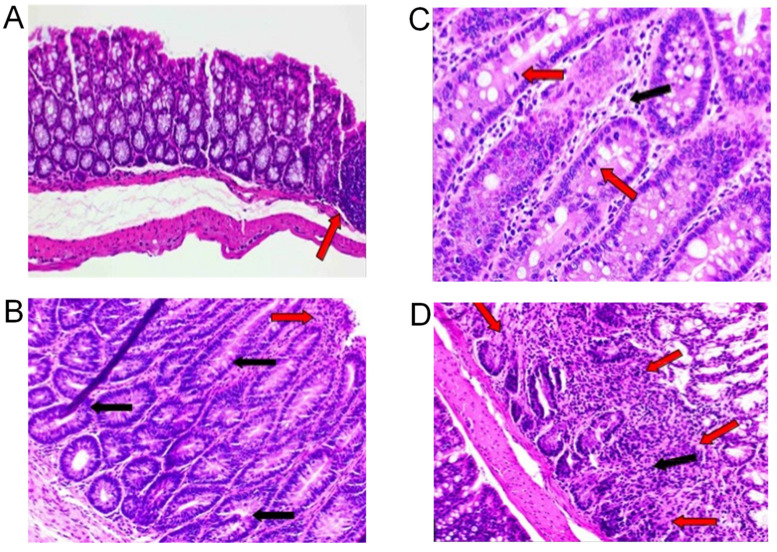
Histological analysis of colonic tissue in C57BL/J6 male mice fed a WD or *n*-6HFD. (**A**) Example of normal section of colon showing the arrangement of crypts in a 10-weeks-old male mouse fed a WD. Arrow points to a mucosal-associated lymphoid tissue (MALT) (20× magnification). (**B**) Representative photomicrograph showing a focus of hyperplasia characterized by elongated and/or tortuous crypts lined with epithelial cells exhibiting increased basophilia and cellular stratification. Black arrows point to the hyperplastic crypts. Red arrow points to the minimally-inflamed surface epithelium associated with mononuclear infiltrates. Slide is from a 16-weeks-old mouse fed the WD (20×). (**C**) Example of hyperplastic crypts showing mitotic figures (red arrows) and mononuclear inflammatory cells (black arrow) that appear to expand the lamina propria around the crypts. The slide is from a 16-weeks-old C57BL/J6 male mouse fed the *n*-6HFD (40×). (**D**) Example of disruption of normal crypt architecture and expansion of the deeper lamina propria by mononuclear inflammatory cells and mild fibrosis (black arrow). Red arrows outline the perimeter of the hyperplastic lesion. The slide is from a 16-weeks-old C57BL/J6 male mouse fed the *n*-6HFD (20×).

Examples of colonic inflammatory and hyperplastic lesions are shown in Figure 3.

At 10 weeks of age, all animals on the WD show normal colon histopathology, and by week 16 of age, only 1/5 animals in the WD group present evidence of chronic-active to chronic inflammation and moderate hyperplasia (Table 1).

**Figure 4:**
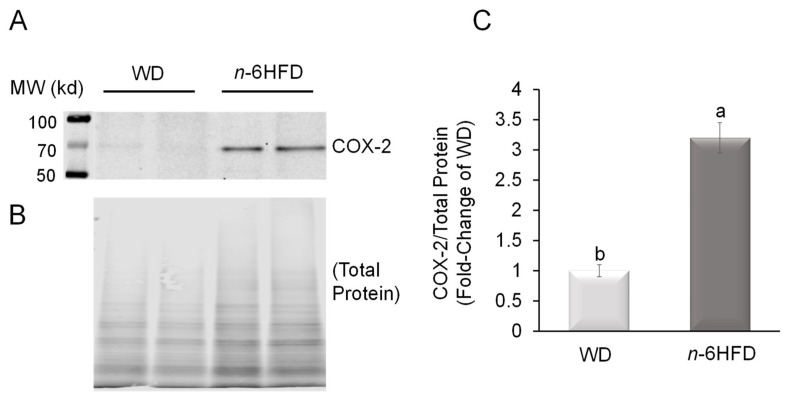
An *n*-6HFD induces COX-2 expression in colonic mucosa. (**A**) Western blot for COX-2 in protein extracts from intestinal mucosa of two representative 16-weeks-old C57BL/J6 male mice fed the WD or *n*-6HFD. (**B**) Total protein staining control. (**C**) Bars are average fold-change levels ± SEM of COX2 protein expression. Means with different letters differ (*p* < 0.05, *n* = 5).

In association with the *n*-6HFD, only one animal presents minimal chronic-active inflammation at 10 weeks of age, but by 16 weeks of age, three out of five animals show abnormal histopathology characterized by hyperplastic lesions and mild fibrosis. Since overexpression of COX-2 is a marker of IBD [22], as a control we assessed the protein level of COX-2 in colonic mucosa (Figure 4A). Animals fed the *n*-6HFD diet exhibit on average a ~3.0-fold increase in the level COX-2 compared to the WD group (Figure 4B). Overall, histopathological and COX-2 expression data indicate that the long-term feeding of the *n*-6HFD increases the prevalence of inflammation and hyperplastic lesions in the colon.

### 2.3. Diversity of the Cecal Microbiota

**Figure 5:**
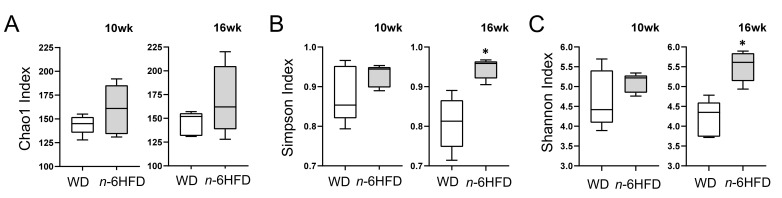
Alpha-diversity of cecal microbiota in C57BL/J6 male mice fed the WD or *n*-6HFD. Box and whisker plots depict alpha-diversity of cecal microbiome in 10-weeks- and 16-weeks-old C57BL/J6 male mice fed the WD or *n*-6HFD estimated using the (**A**) Chao1, (**B**) Simpson, and (**C**) Shannon Index. Differences between the WD and *n*-6HFD were analyzed using the Mann–Whitney U test. Medians with an asterisk (*) differ, *p* < 0.05 (*n* = 5).

Shotgun NGS from DNA of mouse cecal pellets resulted in 52 million total reads passing filter from 20 mice with a mean of 2,643,520 reads/sample and standard deviation of ±193,453. Calculation of alpha-diversity (within sample) of the microbiome using the Chao1 approach does not suggest a significant change in species richness between diets at 10 or 16 weeks of age (Figure 5A). However, both the Simpson (evenness) (Figure 5B) and Shannon (richness and evenness) (Figure 5C) indexes show divergence between *n*-6HFD-fed mice compared to the WD-fed mice at 16 weeks of age, but not at 10 weeks. These analyses denote a time-dependent change in microbiome profile between the two dietary groups.

**Figure 6:**
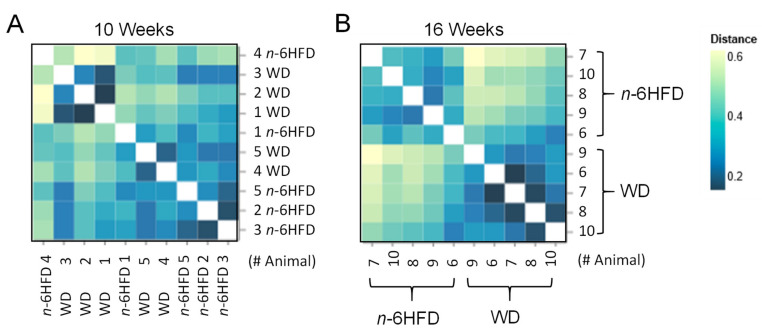
Bray–Curtis Distance Matrix of the cecal microbiota from WD- and *n*-6HFD-fed C57BL/J6 male mice. Bray–Curtis ordination plots based on the bacterial profiles for each mouse at (**A**) 10 weeks and (**B**) 16 weeks. Distance of microbiota between WD and *n*-6HFD-fed mice increases with decreasing color intensity (*n* = 5).

Beta-diversity analysis (Figure 6) reveals at the 10-week time point that the animals in the WD and *n-*6HFD groups are mixed (Figure 6A), whereas the cecal microbiome differs significantly by diet at the 16 weeks of age time-point (*p* < 0.01) (Figure 6B).

**Figure 7:**
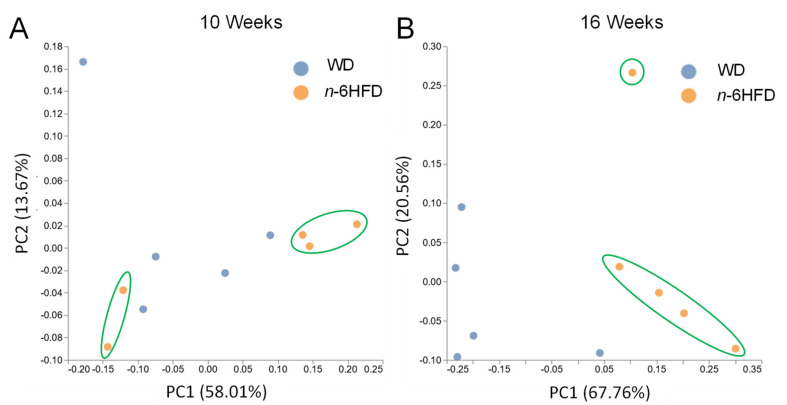
Principal component analysis of the cecal microbiota from WD and *n*-6HFD-fed C57BL/J6 male mice. Diagram axes depict the percent of variance explained by principal component 1 (PC1) and PC2. Plots are based on bacterial relative abundance profiles for each mouse at (**A**) 10 weeks and (**B**) 16 weeks of age. Mice fed *n*-6HFD mice are circled (green) to highlight clustering separation from WD-fed mice.

Compared with samples collected at 10 weeks (Figure 7A), principal component (PC) analysis reveals a clear divergence of cecal samples from WD- compared to *n*-6HFD-fed mice at 16 weeks (Figure 7B), with the PC1 capturing 67.76% of the variation, highlighting the time-dependent divergence due to dietary exposure.

### 2.4. Microbiota Community Composition

**Figure 8:**
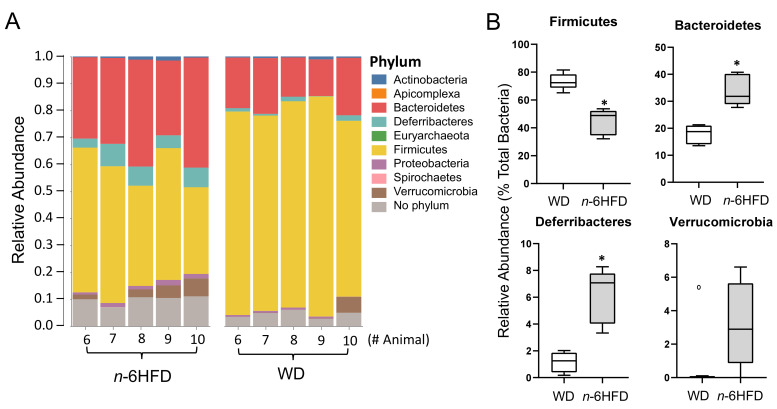
Phyla relative abundances of bacteria in cecal samples of C57BL/J6 male mice at 16 weeks of age. (**A**) Columns represent individual mice fed the WD or *n*-6HFD with bars capturing the relative percent bacterial phylum abundance (% of total). (**B**) Box-and-whisper plots of relative percent abundances (% of total) in cecal samples of WD and *n*-6HFD-fed mice at 16 weeks of age for the phyla *Firmicutes*, *Bacteroidetes*, *Deferribacteres*, *and Verrucomicrobia*. Data were analyzed utilizing the Mann–Whitney U test. Medians with an asterisk (*) differ, *p* < 0.05.

At the 10-week time point, there are no differences in the gut bacterial composition between WD and *n*-6HFD diet (data not shown), although the level of significance (*p* = 0.10) suggests a trend for time-dependent differences. Interestingly, by 16 weeks of age (Figure 8A) we see differences in the relative phyla abundance in cecal samples, with a decrease of *Firmicutes* from 74.2% in the WD group to 44.5% in *n*-6HFD mice, which is paralleled by an enrichment in the relative abundance of *Bacteroidetes* (19% to 32%)*, Deferribacteres* (1.5% to 7.0%), and *Verrucomicrobia* (0.1% to 2.8%) (Figure 8B).

**Figure 9:**
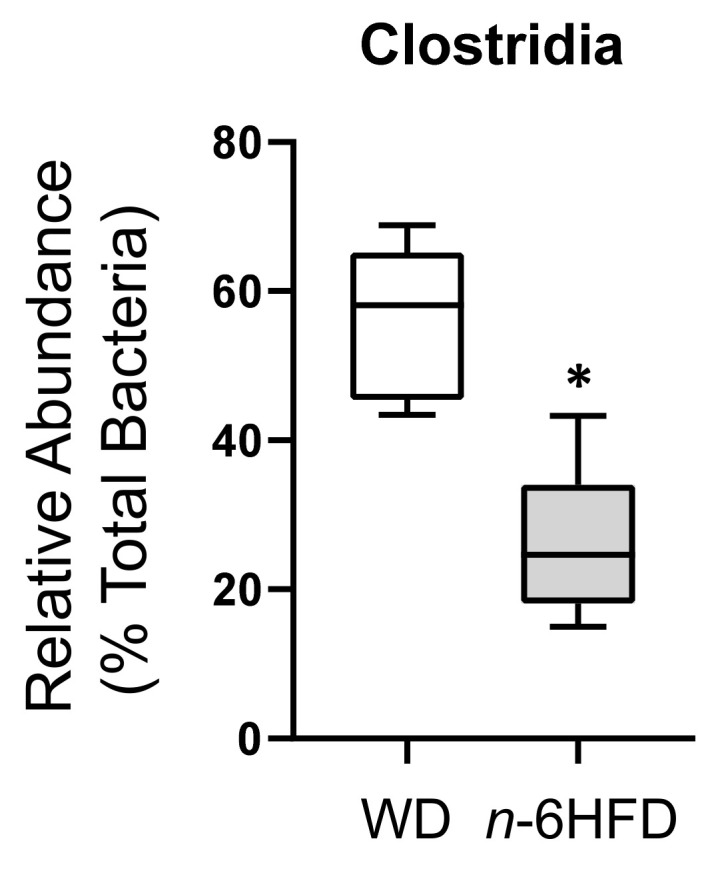
*Chlostridia* bacteria relative abundances in cecal samples of C57BL/J6 male mice at 16 weeks of age. Box and whisker plots are relative percent abundances in cecal samples of WD and *n*-6HFD-fed mice at 16 weeks of age. Data were analyzed utilizing the Mann–Whitney U test. Medians with an asterisk (*) differ, *p* < 0.05.

Given the fact *Firmicutes* represent the majority of the gut microbiome [19] and fat sources influence microbial profile [23], we analyzed the abundance of the class *Chlostridia*, which represent the bulk of this phylum. Results indicate that at 16 weeks of age the *n*-6HFD markedly reduces the percentage of *Chlostridia* from 58.0% to 25% (Figure 9).

**Figure 10:**
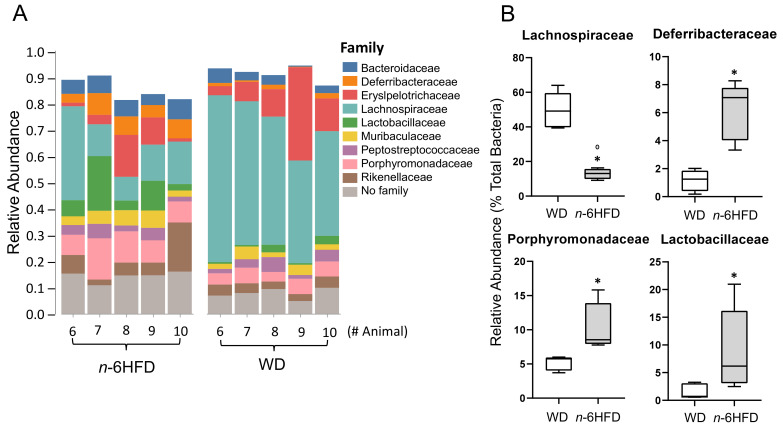
Family relative abundances of bacteria in cecal samples of C57BL/J6 male mice at 16 weeks of age. (**A**) Columns represent individual mice fed the WD or *n*-6HFD with bars capturing the relative percent bacterial family abundance (% of total). (**B**) Box and whisker plots of relative percent abundances (% of total) in cecal samples of WD and *n*-6HFD-fed mice at 16 weeks of age for *Lachnospiraceae, Deferribacteraceae, Porphyromonadaceae, and Lactobacillaceae.* Data were analyzed utilizing the Mann–Whitney U test. Medians with an asterisk (*) differ, *p* < 0.05.

At the family level, in the *n*-6HFD group, there is an increase in the abundance of members of the families *Deferribacteraceae* (1.0% to 7.3%)*, Porphyromonadaceae* (5.2% to 8.0%), and *Lactobacillaceae* (1.0% to 6.0%) (Figure 10A), whereas the relative abundance of *Lachnospiraceae*, which is part of the *Chlostridia* class, decreases from 50.0% to 16%% (Figure 10B).

**Figure 11:**
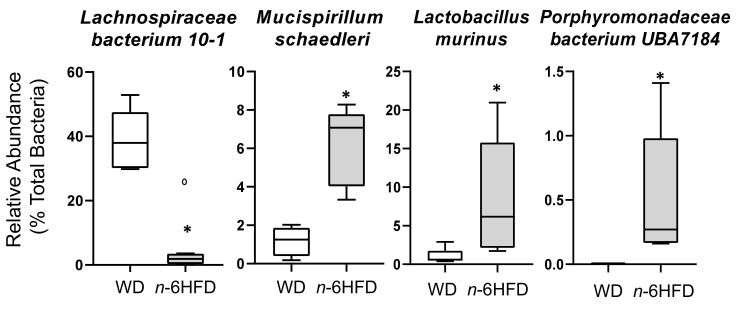
Species-relative abundances of bacteria at 16 weeks of age. Medians with an asterisk (*) differ, *p* < 0.05.

A corresponding downward trend is observed at the species level (Figure 11) for example for *Lachnospiraceae bacterium 10-1* decreasing from 38.0% to 2.0% in the gut flora of *n*-6HFD mice. Conversely, *Mucispirillum schaedleri* and *Lactobacillus murinus* increase from 1.5% to 7.20% and from 1.0% to 6.0%, respectively.

Overall, this taxonomy-based analyses of the cecal microbiota provide important insights into the differential effects of a WD and *n*-6HFD diet on the gut microbiota.

## 3. Discussion

In the U.S., the consumption of *n*-6 fatty acids, primarily from vegetable oils, has increased considerably during the last two decades [5]. Compared to other dietary patterns higher in MUFA and *n*-3 fatty acids [24], diets rich in *n*-6 fatty acids increase the risk of intestinal inflammation [6], colon cancer [7], and obesity [8]. Most rodent studies modeling human consumption of *n*-6HFD have focused on short-term feeding (~2–8 weeks) [13,14,15,16,17,18], whereas rodent gut investigations of microbial ecology modeling human consumption of *n*-6HFD by young adult (8–12 weeks) to adult (>12 weeks) [19] are scarce. Previously, we reported proinflammatory effects of an *n*-6HFD rich in LA on the colon [25]. Here, to model dietary consumption from weaning to adult, male mouse pups were assigned at weaning to either a WD or *n-*6HFD through 10 and 16 weeks of age. The WD provided 58E% from carbohydrates and 27.8%E from fat compared to an isocaloric and isoproteic *n*-6HFD enriched with LA from soybean oil. The WD included fat exclusively from palm oil whereas the *n*-6HFD contained fat exclusively from soybean oil. In agreement with previous reports [14], we show that compared to animals on the WD, at 10 and 16 weeks of age, the *n*-6HFD sustains a double increase in plasma LA and a reduction in palmitoleic and oleic acid accompanied by higher weight gain. This obesogenic response associates with increased colonic COX-2 expression and accumulation of inflammatory and hyperplastic lesions [26].

The gut microbiome plays an important role in the development of IBD [1]. In general, about 90% of the gut microbiome is comprised by *Firmicutes* and *Bacteroidetes* in both humans and mice [19]. We show here that in the WD-fed animals at 16 weeks of age, the *Firmicutes* and *Bacteroidetes* phyla account for ~70% and 20% of the total microbiota, respectively. However, this relative microbial profile changes in the cecal samples of animals fed the *n*-6HFD with loss of *Firmicutes* in favor of *Bacteroidetes*, *Deferricateteres,* and *Verrucomicrobia*. These shifts are accompanied by a drastic reduction in *Clostridia*, a major class of the *Firmicutes* phylum. Higher abundance of *Bacteroidetes* and lower growth of *Firmicutes* was reported in mice fed a diet providing ~38%E as LA from safflower oil [27]. Reduced growth of *Firmicutes* and *Clostridia* combined with gain of *Bacteroidetes* and *Verrucomicrobia* are characteristic microbial shifts of azoxymethane- and dextran sulfate sodium-induced colon inflammation and tumorigenesis [28]. Alterations in gut microbiome may affect the ability of the farnesoid X receptor (FXR) to activate intestinal bile acid (BA) reabsorption [29] and repress COX-2 expression [30]. A reduction in *Clostridia* spp. associates with reduced bile salt hydrolase activity. The latter contributes to deconjugation of BA, leading to intestinal accumulation in rodents of taurine-conjugated β-muricholic acid (TβMCA) (mostly glycine-conjugated in humans), which is a recognized FXR antagonist [31]. Recently, we showed that compared to the WD, the *n*-6HFD induced FXR expression in the small intestine, colon, and liver, as well as increased the levels of primary chenodeoxycholic (CDCA) and cholic (CA) bile acids in cecal samples, and total BAs in the liver. However, the *n*-6HFD diet increased the cecal levels of TβMCA [32]. Therefore, the *n*-6HFD-dependent stimulation of COX-2 expression documented here may be linked to reduced colonization of *Firmicutes*, and more specifically *Clostridia*, and hampered FXR activity due to accumulation of TβMCA in spite of increased FXR expression. This inference is supported by evidence that an HFD rich in saturated palm but low in LA-rich soybean oil favors gut enrichment of *Firmicutes* and *Clostridiales* spp. [16,17].

In human subjects, a decrease in *Firmicutes* is seen in patients with CD [33] and correlates with increased severity of IBD [34]. Enrichment of gut bacteria of the *Bacteroidetes* is more frequent in obese individuals compared to normal-weight individuals [35]. In keeping with these observations, we show that by 16 weeks of age the *n*-6HFD lowers the relative abundance of *Lachnospiracease* (*Clostridia*) and increases the contribution of pathogenic *Deferribacteraceae* and *Porphyromonadaceae* families. Reduced levels of *Lachnospiraceae* and *Clostridiaceae* are seen in UC [33], children, and adolescents with newly diagnosed CD [36]. Conversely, the accumulation of *Deferribacteraceae* and *Porphyromonadaceae* is characteristic of DSS-induced ulcerative colitis [37].

At the species level, 16-weeks-old mice fed the *n*-6HFD display a reduction in *Lachnospiraceae bacterium 10-1* with gain of *Mucispirillum schaedleri* and *Lactobacillus murinus*. Enrichment in *Mucispirillum schaedleri* (Bacteroidetes/*Deferibacteraceae/Bacteroidetes*), a gram-negative anaerobe, is linked to the development of CD-like colitis in rodents [38]. *Mucispirillum schaedleri* appears to well adapt to micro-oxic and redox conditions during gut inflammation [39] and contributes via lipopolysaccharide production to gut inflammation and colon cancer in mothers against decapentaplegic homolog-3-deficient mice [40]. The accumulation of *Lactobacillaceae* and *Lactabacillus murinus* is uniquely interesting since *Lactobacilli* are usually considered as probiotics in the prevention of gut diseases [41]. On the other hand, *Lactobacillus* spp. appear to increase in abundance in the hind gut of mice fed an *n*-6HFD (45%E from fat; *n*-6/*n*-3 = 13), and this correlates with increased plasma concentrations of proinflammatory cytokines, leptin, and TNFα [42]. Similarly, a higher proportion of *Lactobacilli* and lower abundance of *Clostridiales* were documented in mice fed a HFD (60% fat) compared to a high carbohydrate (66%) diet [43]. The increased abundance of *Lactobacillus murinus* correlates with higher serum levels of primary CA and secondary deoxycholic bile acid. Therefore, compared to a WD, an *n*-6HFD rich in LA may alter the relative abundance of *Lactobacillus* spp., favoring the overgrowth of *Lactobacillus murinus* [44].

Human subjects on a high-fiber diet have increased intestinal levels of short chain fatty acids (SCFA) such as butyrate and lower risk of colonic inflammation [45]. Butyrate antagonizes inflammation through repression of nuclear factor *k*-B, a transcription factor that participates in transactivation of *COX2* [46]. Compared to the WD, the *n*-6HFD fed in this study provides higher levels of cellulose (290 vs. 155 g/kg diet) and lower levels of corn starch (87.5 vs. 267 g/kg diet). Strong accumulation of *Lachnospiraceae* is expected on a non-starch polysaccharide (NSP)-based diet [47]. Conversely, cecal pellets from *n*-6HFD show a reduction in *Firmicutes* including *Lachnospiraceae*, which are the main producers of butyrate from NSP in the colon [48]. This microbial imbalance may be due to altered levels of fatty acids in the intestine [43] with LA inhibiting growth of bacteria of the *Firmicutes* phylum (i.e., *Lactobacillus* spp.) [49]. The gut abundance of *Firmicutes* is decreased in HFD-fed mice by LA supplementation, which however sustains the expansion of *Lactobacillaceae* [50]. In summary, these data suggest that an *n*-6HFD rich in LA induces a change in intestinal phylotype through selective inhibition of *Firmicutes,* which include *Lachnospiraceae* and *Clostridia* in spite of relatively higher levels of dietary NSP, while enhancing growth of proinflammatory *Bacteroidetes* [51]. Although due to experimental constraints and to increase focus this study was limited to male mice, we acknowledge that sex-specific factors may influence the microbiota and intestinal inflammatory response to *n*-6HFD. Therefore, comparative studies that model dietary exposure to *n*-6HFD in female models are warranted and represent the subject of ongoing investigations by our group.

## 4. Materials and Methods

### 4.1. Animals, Diets, and Samples Collection

**Figure 12:**
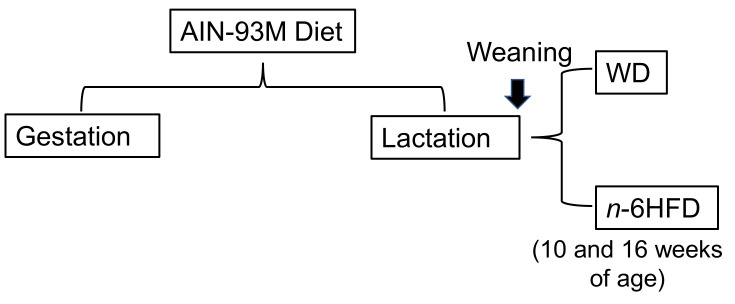
Experimental design and mouse animal model. C57BL/J6 dams were fed a purified diet AIN-93 through gestation and lactation. At weaning, male mice were assigned to either a Western diet (WD) or an *n*-6 high-fat diet (*n*-6HFD) through 10–16 weeks of age.

Breeder pairs of C57BL/6J mice (The Jackson Laboratory, Bar Harbor, ME, USA) were fed AIN93M purified diet (Envigo/Teklad, Indianapolis, IN, USA). Dams were maintained on this diet during gestation and trough lactation. At weaning, male mice were assigned to either a WD containing 27.8%E from fat (11% palm oil, by weight) or an isocaloric *n*-6HFD containing 50.3%E from fat (20% soybean oil by weight) through 10 or 16 weeks of age (Figure 12).

The WD and *n*-6HFD were formulated to be isocaloric (kcal/g) by adjusting the percentage of carbohydrates (corn starch) and fiber (cellulose), whereas the percentage of protein, sugar, vitamins, and minerals remained unchanged between the two diets (Table 2 and Table 3).

Litters were allowed chow and water ad libitum, and body weights were measured weekly. At the end of the experimental periods, animals were sacrificed and blood, cecal pellets, and colonic mucosa were collected. Plasma was extracted from heparinized blood after centrifugation at 14,000 rpm for 10 min at 4 °C and stored frozen. Collection of the mucosal cells was performed as previously described [51]. Scraped colonic cells were rinsed with PBS and then separated by centrifugation at 14,000 rpm for 10 min at 4 °C.

### 4.2. Western Blot Analysis

Western blotting was performed as previously described [52]. Briefly, total protein was extracted from colonic mucosa by suspending ~30 mg of tissue in Pierce RIPA Buffer (Thermo Fisher Scientific, Waltham, MA, USA) containing a 1% concentration of protease inhibitor (VWR International, Radnor, PA, USA). Samples were incubated on ice for 45 min with occasional vortexing. After incubation, samples were centrifuged at 16,000× *g* for 10 min at 4 °C to separate cell debris from the protein lysate. Protein concentration was determined using the Nanodrop1000 Spectrophotometer (Thermo Fisher Scientific). Samples were prepared for polyacrylamide gel electrophoresis by heating 100 μg of protein (normalized with water) at 65 °C for 4 min. Then, an equal volume of Laemlli buffer (Biorad, Hercules, CA, USA) containing 1% β-mercaptoethanol was added. This mixture was heated in a water bath for 4 min, cooled to room temperature for 4 min, then centrifuged at 10,000× *g* for 30 s. Proteins were separated on Novex Wedgewell 4–12% tris-glycine gels (Invitrogen, Waltham, MA, USA) using a constant voltage (100 V) for ~75 min. Proteins were transferred to nitrocellulose membranes (Amersham, Amersham, UK) using the Mini Blot Module and Mini Gel Tank (Invitrogen) wet-transfer system. Transfer was conducted in tris-glycine transfer buffer (15% methanol) at 15 V for 45 min. Blocking was performed for 1 h at room temperature with a 1% casein blocking buffer dissolved in tris-buffered saline containing 1% NaCl. Polyclonal antibodies against COX-2 (Novus Biologicals, Littleton, CO, USA) and anti-rabbit secondary antibodies (LI-COR Biosciences, Lincoln, NE, USA) were diluted in 1% casein blocking buffer dissolved in TBS + 0.01% tween, and primary incubations were carried out overnight at 4 °C. Then, nitrocellulose membranes were incubated with a secondary antibody for 1 h at room temperature. Immunocomplexes were detected by near-infrared using an Odyssey CLx scanner (LI-COR Biosciences). Staining of total protein was used as a control for signal quantitation [53], which was performed using Revert Total Protein Stain and ImageStudio Lite (LI-COR Biosciences).

### 4.3. Sequencing of Microbiota in Cecal Pellets

DNA was extracted from mouse cecal pellets preserved in DNA/RNA Shield (Zymo Research, Irvine, CA, USA) utilizing Qiagen’s PowerFecal Pro DNA kit (Qiagen, Hilden, Germany) following manufacturer’s protocol. Extracted DNA was quantified with the ONE dsDNA QuantiFluor Dye System on Quantus Fluorometer (Promega, Madison, WI, USA). Following quantitation, purified sample DNA was normalized for downstream tagmentation and library fabrication utilizing Nextera DNA Flex kit (Illumina, San Diego, CA, USA). Prepared and indexed libraries were then pooled for next generation sequencing (NSG) with NextSeq 500/550 Mid-Output Kit v2.5 (300 cycles) (Illumina).

### 4.4. Plasma Fatty Acid Profile

Mouse plasma was subjected to fatty acid methyl ester derivatization following established protocols [54]. Briefly, 100 µL of plasma was added to 100 µg of C17 (triheptadecanoin) internal standard. Then, samples underwent saponification to liberate fatty acids, which were derivatized utilizing 12% boron trifluoride at 100 °C degrees for 5 min. A panel of 37 fatty acids was separated on a J&W DB20-FastFAME column and analyzed utilizing an Intuvo 9000 gas chromatography and flame ionization detection (Agilent, Santa Clara, CA, USA) [55]. Data were expressed as percent of total fatty acids. Percent total calculations contained only lipids that exhibited a signal four times greater than baseline.

### 4.5. Colon Histopathology

Hematoxylin and eosin-stained colon sections from animals were examined microscopically. Sample blind scoring of colonic inflammation was performed by a certified pathologist (Integrated Laboratory Systems, Research Triangle Park, NC, USA) according to previous guidelines [56]. When reported, inflammation was accompanied by disruption of mucosal architecture and variable expansion of the lamina propria with mononuclear inflammatory cells, with or without fibrosis. Based on the presence or absence of neutrophils and/or evidence of fibrosis, inflammation was scored as chronic-active or chronic, respectively. Inflammation associated with hyperplasia of the crypts was examined for changes in basophilia, nuclear stratification, and mitoses. Hyperplastic lesions were classified as focally extensive and scored as preneoplastic lesions.

### 4.6. Microbiome and Statistical Analyses

Generated and processed text-based format for storing both a biological sequence and its corresponding quality scores were passed through One Codex’s metagenomic pipeline for taxonomic classification and microbiome analysis. The One Codex Database consists of ~114K complete microbial genomes (One Codex, San Francisco, CA, USA). During processing, reads were first screened against the human genome, then mapped to the microbial reference database using a k-mer based classification. Individual sequences (NGS read or contig) were compared against the One Codex Database (One Codex) by exact alignment using *k*-mers, where *k* = 31 [57,58]. Based on the relative frequency, unique *k*-mers were filtered to control for sequencing or reference genome artifacts. The relative abundance of each microbial species was estimated based on the depth and coverage of sequencing across every available reference genome. Secondary analyses of the taxonomic classifications, including alpha- and beta- diversity calculations, were all performed using the One Codex Library (One Codex). Statistical analyses of alpha-diversity and differential abundance were performed using the Mann–Whitney U test implemented in the SciPy software library for scientific computing. Statistical analysis of beta-diversity was performed using a permutational multivariate analysis of variance, as implemented in the Scikit-bio software library [59]. Beta-diversity was calculated as the pairwise Bray–Curtis dissimilarity using the species-level relative abundance vectors obtained from One Codex. All secondary analyses were performed in a Jupyter Notebook on the One Codex platform (One Codex). Box and whisker plots were created utilizing the GraphPad Prism software package version 8.3 (Graph-Pad Software, San Diego, CA, USA) [60]. Differences related to WD and *n*-6HFD were analyzed by 1-way ANOVA for body weight and COX-2 expression. For plasma fatty acids profile, we used a 2-way ANOVA mixed-model to account for the interrelationships between the WD and *n*-6HFD at the 10 and 16 week of age time-points and corrected for differences in group size. Significant differences (*p* < 0.05) were determined using Tukey’s HSD test. Statistical analysis was performed using Prism (Graph-Pad Software).

## 5. Conclusions

In recent decades, one of the recommendations for Americans and Western populations has been increased intake of PUFA, resulting in higher intake of *n*-6 over *n*-3 fatty acids [61]. Our findings suggest that higher consumption of *n*-6 LA over those normally consumed through a typical American WD shift unfavorably the gut microbiome towards a pathobiontic profile. Future studies are needed to examine the effects of an *n*-6HFD in female preclinical models and human subjects. Also, the impact of different fatty acids (i.e., SFA, MUFA, *n*-6 vs. *n*-3 PUFA) and interactions with types and amounts of dietary carbohydrates on colonic inflammation and microbiome profile represent important areas of investigation.

## Figures and Tables

**Table 1:** Observed Colonic Inflammatory Events.

Experimental Diets	WD		*n*-6HFD	
Experimental Periods	10 Weeks (*n* = 5)	16 Weeks (*n* = 5)	10 Weeks (*n* = 5)	16 Weeks (*n* = 5)
Number of animals with normal histopathology	5	4	4	2
Number of animals with colonic events: Mucosal fibrosis:				
Minimal	0	0	0	0
Mild	0	0	0	1
Moderate	0	0	0	0
Marked	0	0	0	0
Mucosal hyperplasia:				
Minimal	0	0	0	0
Mild	0	0	0	0
Moderate	0	1	0	0
Marked	0	0	0	1
Inflammation, chronic-active:				
Minimal	0	1	1	2
Mild	0	0	0	0
Moderate	0	0	0	0
Marked	0	0	0	0
Inflammation, chronic:				
Minimal	0	1	0	0
Mild	0	0	0	1
Moderate	0	0	0	0
Marked	0	0	0	0

**Table 2:** Nutrient Composition of Diets ^a^.

Diet Formula	AIN-93M (g/Kg)		WD (g/Kg)		*n*-6HFD (g/Kg)	
Casein	140.0		140.0		140.0	
L-Cystine	1.8		1.8		1.8	
Corn Starch	465.7		267.5		87.5	
Maltodextrin	155.0		155.0		155.0	
Sucrose	100.0		100.0		100.0	
Soybean Oil	40.0				200.0	
Palm Oil			110.0			
Cellulose	50.0		155.0		290.0	
Mineral Mix, AIN-93M-MX (94049)	35.0		35.0		35.0	
Mineral Mix, AIN-93-VX (94047)	10.0		10.0		10.0	
Choline Bitartrate	2.5		2.5		2.5	
TBHQ, Antioxidant	0.01		0.02		0.04	
**Nutrient Composition**	**% Weight**	**% Kcal**	**% Weight**	**% Kcal**	**% Weight**	**% Kcal**
Protein	12.4	13.7	12.4	13.7	12.4	13.7
Carbohydrate	68.3	75.9	52.8	58.4	32.3	35.9
Fat	4.1	10.3	11.1	27.8	20.1	50.3
Energy (Kcal/g)	3.6		3.6		3.6	

^a^ Values are calculated from ingredient analysis or manufacturer data (Teklad Laboratory). WD = Western diet; *n*-6HFD = diet enriched with *n*-6 fatty acids; TBHQ = tertiary butyl-hydroquinone.

**Table 3:** Fatty Acid Composition of Diets ^a^.

Diet Formula	AIN-93M (g/Kg) %	WD (g/Kg) %	*n*-6HFD (g/Kg) %
Total fat (g/Kg)	41.4	111.4	201.4
SFA (g/Kg)	6.0 15.1	56.2 51.3	30.0 15.1
MUFA (g/Kg)	9.4 23.5	42.7 39.0	48.8 23.5
PUFA (g/Kg)	24.5 61.4	10.7 9.7	122.4 61.4

^a^ Values are from manufacturer’s data (Teklad Laboratory). WD = Western diet; *n*-6HFD = diet enriched with *n*-6 fatty acids.

## Data Availability

The datasets generated during and/or analyzed during the current study are available from the corresponding author on reasonable request.

## Supplementary Materials

The following are available online at https://www.mdpi.com/article/10.3390/ijms22136919/s1.

## Abbreviations

CA	cholic acid
CD	Chron’s disease
COX-2	cyclooxygenase-2
FXR	farnesoid X receptor
HFD	high fat diet
IBD	inflammatory bowel disease
LFD	low fat diet
LA	linoleic acid
MUFA	monounsaturated fatty acids
NGS	next generation sequencing
*n*-6HFD	*n*-6 high fat diet
NSP	non-starch polysaccharide
PC	principal component
%E	percent energy
PUFA	polyunsaturated fatty acids
SFA	saturated fatty acids
TβMCA	taurine-conjugated β-muricholic acid
UC	ulcerative colitis
WD	Western diet
