# Supplementary figures and images for: n-6 High Fat Diet Induces Gut Microbiome Dysbiosis and Colonic Inflammation
Source: Int J Mol Sci. 2021 Jun 28;22(13):6919. doi: 10.3390/ijms22136919 (PMC8269411; doi:10.3390/ijms22136919)

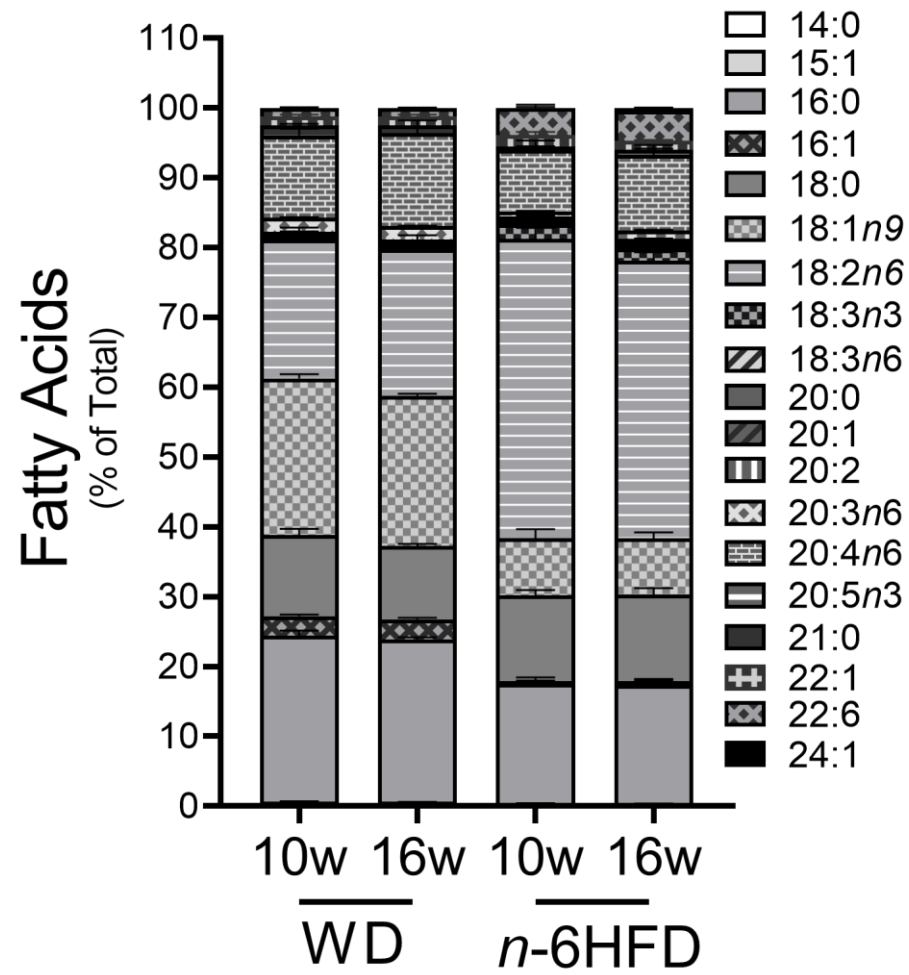

Supplement: Supplementary file 1 [file ijms-22-06919-s001.zip › ijms-1197978-supplementary.pdf]
